# Comparison of the effectiveness of nebulizer treatment applications in acute exacerbation of chronic obstructive pulmonary disease: a randomized controlled trial

**DOI:** 10.1590/1806-9282.20240861

**Published:** 2024-12-02

**Authors:** Buğra Kerget, Erdal Tekin, Gizem Çil, Kadir Çelik, Alperen Aksakal

**Affiliations:** 1 Ataturk University, School of Medicine, Department of Pulmonary Diseases – Erzurum, Turkey.; 2 Ataturk University, School of Medicine, Department of Emergency Medicine – Erzurum, Turkey.

**Keywords:** COPD, Disease exacerbation, Nebulizers, Pulmonary function test

## Abstract

**OBJECTIVE:**

In addition to oxygen support, bronchodilator therapy constitutes the most essential treatment step in patients with chronic obstructive pulmonary disease. Our study aimed to compare the effectiveness of nebulizer treatments in patients who presented to the emergency department with acute exacerbation of chronic obstructive pulmonary disease.

**METHODS:**

Our study included 60 Group E chronic obstructive pulmonary disease patients admitted to the emergency department due to acute exacerbation of chronic obstructive pulmonary disease between June and September 2023 (NCT: 06178068). Patients were randomized as follows: Group 1: jet nebulizer treatment; Group 2: dry air nebulizer treatment; and Group 3: classic nebulizer treatment.

**RESULTS:**

While an increase in forced vital capacity (FVC) and forced expiratory volume 1 (FEV1) levels was observed in Group 1 patients (in the order of p=0.009 and 0.007), a decrease in residual volume (RV) and total lung capacity (TLC) levels was observed (p=0.02 and 0.05, respectively). At the same time, an increase in FEV1 levels was observed in Group 2 and 3 patients (p=0.04 and 0.04, respectively). A decrease was observed in RV and TLC levels (p=0.02, 0.05, 0.02, and 0.04, respectively). When comparing the respiratory function test parameters at the beginning and on the fifth day of treatment, FEV1 and peak expiratory flow 25/75 (PEF25/75) levels were higher in Group 1 patients than in Group 2 and 3 patients (p=0.01 and 0.02, respectively).

**CONCLUSION:**

In chronic obstructive pulmonary disease patients, jet nebulizer treatment is more effective regarding bronchodilator activity than other nebulizer treatments. However, dry air nebulizer treatment may be preferred in chronic obstructive pulmonary disease patients because it is easily applicable and sterilizable.

## INTRODUCTION

Chronic obstructive pulmonary disease (COPD) is a heterogeneous condition caused by an airway (bronchitis/bronchiolitis) or alveolar (emphysema) abnormality characterized by chronic respiratory symptoms (dyspnea, cough, and sputum). It progresses with persistent and often progressive airway obstruction^1^. In COPD, which progresses with high mortality, both non-pharmacological treatments and pharmacological treatments are applied. These treatments have an essential role in slowing down the progressive course of the disease^2^. However, despite the treatments applied to COPD patients, infections, outdoor air pollution, improper use of inhaler treatments, or comorbidities may cause acute exacerbation of COPD^3^.

Exacerbation in the GOLD 2023 report: It is defined as the worsening of symptoms of cough, sputum, and shortness of breath, which may be accompanied by tachypnea and tachycardia, within the last 14 days as a result of increased local and systemic inflammation due to infection, air pollution, or other exposure^4^. Bronchodilator therapy, along with increasing oxygenation, forms the basis of treatment in patients hospitalized due to exacerbation. It has been observed that bronchodilator therapy in nebulizer form is more effective in patients presenting with exacerbations^4^. Providing oxygen support with an oxygen saturation of 88–92% has a key role in the treatment of patients hospitalized due to acute exacerbation. After oxygen therapy is initiated, arterial blood gas should be monitored until it is ensured that adequate oxygen supply is provided without carbon dioxide (CO_2_) retention and a shift to acidosis^5^. The possibility that nebulizer treatment with high-flow oxygen may cause carbon dioxide retention during bronchodilator treatment has brought the use of jet nebulizer bronchodilator treatment to the agenda in patients with COPD. The high gas flow produced by a jet nebulizer electric compressor converts the particles into respirable size with a 1–5 μm diameter and settles them in the tracheobronchial system^6^.

Dry air nebulizer devices use dry air from the central system in hospitals. This system, which converts bronchodilator drugs into nebulizers by creating high currents, works like classic nebulizers without using an electrical system and can create a higher flow rate than jet nebulizer devices. This procedure, which can be applied to COPD patients without giving high-flow oxygen, prevents carbon dioxide retention. There is no study in the literature comparing the bronchodilator effectiveness of dry air, classic nebulizer, and jet nebulizer, and we aimed to compare the effectiveness of these three treatment modalities in our study.

## METHODS

### Study design

Patients over 40 years admitted to the emergency department for acute COPD exacerbation between June and September 2023, who completed treatment and were admitted to the chest diseases service, were included. G*Power analysis determined a sample size of 13 patients per group (α error=0.05, 1–β error=0.95, d=0.80). The study followed the Declaration of Helsinki’s ethical principles and received local ethics committee approval. All participants provided written informed consent (ClinicalTrials Number: 06178068).

### Study population

Bronchodilator treatment was administered with a jet nebulizer for 2 h at 20-min intervals to patients admitted for acute COPD exacerbation. A pulmonary function test (PFT) was conducted to confirm COPD diagnosis and assess basal respiratory function. Eligible patients were randomized into three groups of 20 using Microsoft Excel^®^. Group 1 received bronchodilator treatment every 4 h with a jet nebulizer; Group 2 received treatment every 4 h with a dry air nebulizer; and Group 3 with a classic nebulizer. All other COPD acute exacerbation treatments followed the GOLD 2023 guideline^7^.

#### Exclusion criteria

Patients were excluded if they had conditions contraindicating pulmonary function testing (recent MI, pulmonary embolism, cerebral aneurysm, active hemoptysis, pneumothorax, nausea, vomiting, and recent surgeries), mental retardation, lack of cooperation, pneumonia with acute COPD exacerbation, pulmonary edema due to heart failure, or interstitial lung disease with COPD.

#### Application of pulmonary function test

Protective equipment was used by personnel during PFT. Patients’ age, height, and weight were measured beforehand. Smoking, alcohol consumption, heavy exercise, restrictive clothing, and heavy meals were avoided before testing. A body temperature, pressure, water vapor saturated (BTPS) correction was made based on room air and pressure. The maneuver was explained to the patient, who then performed three acceptable spirograms. Tests that met ATS/ERS 2019 criteria were included. Spirometry was conducted by the same technician using the Cosmed Q-Box Body Plethysmography device^8^.

### Method of application of nebulizer treatments

The Respirox^®^ RNEB-18 device was used for pre-randomization and Group 1 maintenance bronchodilator treatments, calibrated to nebulize at 6 L/min. Group 2 used a HAVELSAN^®^ EHSİM dry air nebulizer, while Group 3 used a classic nebulizer compatible with the hospital’s central oxygen system, both of which were manually adjustable and set to 6 L/min.

### Statistical analysis

IBM SPSS Statistics for Windows version 24.0 (IBM Corp., Armonk, NY) was used for statistical analyses. The distribution of continuous variables was tested for normality using the Shapiro-Wilk and Kolmogorov-Smirnov tests. The results indicated a non-normal distribution, and the data were analyzed using non-parametric tests. The Kruskal-Wallis test was used to compare the laboratory and PFT data of the groups. A Mann-Whitney U test was used to compare laboratory and PFT data between groups. A Wilcoxon test was used to evaluate the changes in PFT and laboratory parameters at the beginning and 5th day of treatment. p<0.05 were considered statistically significant.

## RESULTS

The average age of the patients in our study was 68.3±14.4 years. The mean age of Group 1 patients was 65.4±13.3; Group 2 patients was 66.5±16.1; and Group 3 patients was 65.5±18.1. Additionally, 58.9% (n=23) of the patients were male. In the statistical analysis of the groups according to gender and age, it was observed that there was no significant difference between the groups (p=0.33 and 0.78, respectively).

It was observed that the most common comorbidity in patients after COPD was hypertension (n=35). Hypertension was followed by diabetes mellitus (n=11) and coronary artery disease (n=10).

The evaluation of PFT and blood gas analyses performed after bronchodilator treatment was given in the emergency department without randomizing the groups, and it is given in Tables 1 and 2. In the statistical analysis performed between the initial respiratory function test and blood gas parameters of the groups, it was observed that none of the parameters showed a statistically significant difference (p>0.05 for all). In the respiratory function test analysis of the groups at the beginning of treatment and on the fifth day of treatment, an increase in forced vital capacity (FVC) and forced expiratory volume 1 (FEV1) levels was observed in Group 1 patients (in the order of p=0.009 and 0.007). In contrast, a decrease in residual volume (RV) and total lung capacity (TLC) levels was observed (in the order of p=0.02 and 0.05). While an increase in FEV1 level was observed in Group 2 patients (p=0.04), a decrease was observed in RV and TLC levels (p=0.02 and 0.05, respectively). In Group 3 patients, an increase in FEV1 level was observed (p=0.04), but a decrease in RV and TLC levels was observed (p=0.02 and 0.04, respectively).

**Table 1:** Comparison of pulmonary function test parameters of the groups at the beginning and fifth day of treatment.

Group 1 (Jet nebulizer group) (n=20)	At the beginning of treatment	Fifth day of treatment	p
FVC (%)	73.5 (40–90)	90.5 (40–137)	0.009
FEV1 (%)	38 (17–61)	41 (23–93)	0.007
FEV1/FVC (%)	43 (30–62)	45 (35–65)	0.12
RV (%)	220 (140–415)	154 (55–275)	0.02
TLC (%)	138 (85–228)	123.5 (94–256)	0.05
**Group 2 (Dry air nebulizer group) (n=20)**	At the beginning of treatment	Fifth day of treatment	p
FVC (%)	73 (50–89)	73 (38–90)	0.44
FEV1 (%)	35 (22–63)	37.5 (23–65)	0.04
FEV1/FVC (%)	44 (28–64)	45 (30–66)	0.34
RV (%)	269 (96–465)	154 (55–275)	0.02
TLC (%)	162.5 (57–236)	104 (52–208)	0.05
**Group 3 (Classic nebulizer group) (n=20)**	At the beginning of treatment	Fifth day of treatment	P
FVC (%)	72.8 (45–91)	74 (46–92)	0.5
FEV1 (%)	36 (18–62)	38 (22–65)	0.04
FEV1/FVC (%)	41 (28–61)	44 (29–64)	0.23
RV (%)	244 (130–405)	150 (90–246)	0.02
TLC (%)	150 (66–210)	105 (64–202)	0.04

**Table 2:** Comparison of arterial blood gas parameters of the groups at the beginning and fifth day of treatment.

Group 1 (Jet nebulizer group) (n=20)	At the beginning of treatment	Fifth day of treatment	p
Ph	7.39 (7.35–7.47)	7.4 (7.38–7.49)	0.6
SO2 (%)	81 (69–90)	88 (74–95)	0.01
pO2 (mm_Hg)	48 (37–59)	61 (49–88)	0.03
pCO2 (mm_Hg)	46.5 (33–58)	42 (27–53)	0.07
HCO3 (mEq/L)	28.2 (19–33)	36.5 (21–34)	0.3
**Group 2 (Dry air nebulizer group) (n=20)**	At the beginning of treatment	Fifth day of treatment	p
Ph	7.38 (7.35–7.46)	7.4 (7.36–7.49)	0.09
SO2 (%)	85 (58–90)	90 (85–95)	0.01
pO2 (mm_Hg)	55.5 (34–72)	59 (41–80)	0.03
pCO2 (mm_Hg)	46 (30–61)	44.5 (27–61)	0.23
HCO3 (mEq/L)	26 (21–41)	25.5 (21–36)	0.7
**Group 3 (Classic nebulizer group) (n=20)**	At the beginning of treatment	Fifth day of treatment	p
Ph	7.38 (7.35–7.45)	7.36 (7.34–7.43)	0.07
SO2 (%)	86 (64–89)	89 (85–94)	0.05
pO2 (mm_Hg)	53 (37–60)	56 (38–79)	0.05
pCO2 (mm_Hg)	45 (32–59)	47 (32–64)	0.08
HCO3 (mEq/L)	26 (22–43)	27 (23–45)	0.67

In comparing arterial blood gas parameters before treatment and on the fifth day of treatment, SO_2_ and pO_2_ levels were observed in Group 1, 2, and 3 patients (p=0.01, 0.03, 0.01, 0.03, 0.05, and 0.05, respectively). In evaluating pCO_2_ levels, no significant difference was observed in all groups before and after treatment. However, it was observed that the parameter, which decreased with treatment in Group 1 and 2 patients, increased in Group 3 patients.

A comparison of changes in PFT parameters between nebulizer treatments is shown in Table 3. Accordingly, it was observed that FEV1 and peak expiratory flow 25/75 (PEF25/75) levels differed significantly between the groups (p=0.01 for both). In the comparison between the groups, it was observed that this change was higher in Group 1 patients than in Group 2 and 3 patients (p=0.01 and 0.02, respectively), while there was no significant difference between Group 2 and 3 patients (p=0.35).

**Table 3:** Comparison of the change in pulmonary function test parameters of the groups after 5 days of treatment.

	Group 1 (n=20)	Group 2 (n=20)	Group 3 (n=20)	P
ΔFEV1 (%)	5.5 (1 to 48)	2 (-5 to 10)	3 (-2 to 9)	0.01
ΔRV (%)	-12.5 (-93 to 107)	-50 (-218 to 35)	-35 (-160 to 20)	0.12
ΔTLC (%)	-12 (-36 to 38)	-16 (-175 to 118)	-17 (-140 to 34)	0.38
ΔPEF25/75 (%)	13 (4 to 19)	8 (2 to 10)	7 (2 to 12)	0.01

## DISCUSSION

Our study observed that jet nebulizer, dry air, and classic nebulizer treatments caused an increase in FEV1 levels and a decrease in RV and TLC levels. It was also observed that all nebulizer treatments increased the saturation and pO_2_ parameters. In comparing the bronchodilator effect of bronchodilator treatment on the airways, it was observed that the bronchodilator effect of jet nebulizer treatment in small airways was better than the other two treatments.

Bronchodilator therapy is an integral part of the treatment in acute exacerbations of COPD. Dynamic hyperinflammation during acute exacerbation is the most crucial cause of exertional dyspnea and respiratory distress in people. Bronchodilator treatments provide a regression of dynamic hyperinflammation in patients presenting with acute exacerbation^9^. Studies evaluating the application of bronchodilator therapy in patients presenting with acute exacerbation of COPD have found different results between metered dose inhaler (MDI) therapy and nebulizer therapy^10,11^. However, the reality is that nebulizer drug treatment is more costly than MDI treatment. In addition, due to the way nebulizer treatment is applied, it can be more comfortable in dyspneic patients, as it provides bronchodilator treatment independent of effort^12-14^.

Giving high-flow oxygen therapy to patients presenting with acute exacerbation of COPD may cause hypercapnic respiratory failure^15^. During the classical application of nebulizer treatment, high-flow oxygen had to be given in order for nebulization to occur. Jet nebulizer technology has been developed to both administer bronchodilator therapy and avoid hypercapnia^9^. Jet nebulizers nebulize bronchodilator therapy during the patient’s inspiration and prevent wasting bronchodilator therapy during expiration^13,16^. The most important disadvantage of jet nebulizer treatments is that they need to be cleaned very carefully, and they require electricity to work^17,18^.

Dry air nebulizer treatment is integrated into hospital central systems and can create varying flow levels to convert bronchodilator treatment into a nebulizer. The manufacturer recommends a flow rate of at least 6 L/min. Unlike jet nebulizers, dry air nebulizers do not require high-flow oxygen therapy and are easier to clean. However, converting bronchodilator treatment into a nebulizer during both inspiration and expiration may result in lower bronchodilator activity at the same dose. This treatment has recently been developed in our country, and its effectiveness and potential disadvantages have been observed based on our experience.

In our study, when evaluating the effectiveness of nebulizer treatment applications on respiratory function test parameters, it was observed that all treatment applications caused an increase in FEV1 levels. However, in the analysis where the change in FEV1 levels was evaluated together with the shift in PEF25/75, it was observed that Group 1 patients had more successful results than the other groups. In jet nebulizer treatment, the bronchodilator treatment becomes a nebulizer during inspiration and decreases during expiration, ensuring that the drug passes into the lungs more effectively and more. In other groups, continuing nebulizer treatment independently of inspiration and expiration causes this situation to decrease. Our study observed better bronchodilator treatment effectiveness in patients receiving jet nebulizer treatment, which may be due to this situation. In the evaluation of blood gas parameters, it was observed that all nebulizer treatment methods provided a significant increase in both saturation and partial oxygen levels. Even though there was no statistically significant difference in partial carbon dioxide levels, it was observed that the level decreased in Group 1 and 2 patients and increased in Group 3 patients. This may have developed primarily due to respiratory depression caused by high oxygen therapy applied to COPD patients. In addition, dry air nebulizer treatment has bronchodilator effectiveness. It does not cause carbon dioxide retention like jet nebulizer treatment, indicating that it may be an alternative treatment method in patients presenting with hypercapnic respiratory failure.

The treatment dose may need to be increased in order for classic nebulizer treatment and dry air nebulizer treatments to reach the lungs as much as jet nebulizer treatment. To not disrupt standardization in our study, all patients were administered the same amount of bronchodilator treatment. However, studies that measure the lung accumulation levels of all nebulizer treatments may be more helpful in evaluating the effectiveness of nebulizer treatments and cost analysis.

As a result, jet nebulizer treatments are still one of the most effective methods in applying bronchodilator treatment in obstructive lung diseases. However, problems encountered in sterilizing these devices and the need for electricity may pose a disadvantage. The application of dry air nebulizer therapy can be as effective as classic nebulizer treatments without causing carbon dioxide retention in patients with COPD. The ease of sterilization and lack of electricity are other advantages. For this reason, dry air nebulizer treatment can be used as an alternative method in departments with high patient circulation.
